# Editorial: Circadian rhythm in obesity

**DOI:** 10.3389/fendo.2024.1387889

**Published:** 2024-03-11

**Authors:** Olga Pivovarova-Ramich, Steven K. Malin

**Affiliations:** ^1^ Research Group Molecular Nutritional Medicine and Department of Human Nutrition, German Institute of Human Nutrition Potsdam-Rehbruecke, Nuthetal, Germany; ^2^ German Center for Diabetes Research (DZD), München-Neuherberg, Germany; ^3^ Department of Endocrinology and Metabolism, Charité – Universitätsmedizin Berlin, Corporate Member of Freie Universität Berlin and Humboldt-Universität zu Berlin, Berlin, Germany; ^4^ Department of Kinesiology and Health, Rutgers University, New Brunswick, NJ, United States; ^5^ Division of Endocrinology, Metabolism and Nutrition, Rutgers University, New Brunswick, NJ, United States; ^6^ New Jersey Institute for Food, Nutrition and Health, Rutgers University, New Brunswick, NJ, United States; ^7^ Institute of Translational Medicine and Science, Rutgers University, New Brunswick, NJ, United States

**Keywords:** circadian rhythm, circadian clock, obesity, shift workers, circadian disruption, metabolism

Circadian rhythm plays a crucial role in orchestrating a wide range of physiological processes, including endocrine functions, glucose, lipid, and protein metabolism, inflammatory processes, as well as even the gut microbiome (1, 2). Adequate function of the circadian clock is key to maintaining metabolic health, whereas obesity and obesity-associated metabolic diseases, such as type 2 diabetes and cardiovascular disease, often demonstrate disturbed circadian rhythmicity (3). Indeed, while alterations in light exposure can disrupt circadian rhythms through changes in the central clock of the hypothalamic suprachiasmatic nucleus, it is important to note that different timing of food intake or exercise across the 24 hour period (e.g., during the night vs. day) as well as disruptions in the sleep-wake cycle as seen in shift-workers can negatively impact mental health as well as increase body weight and metabolic disease risk (4, 5).

Despite the body of evidence highlighting the tight connections between circadian physiology and obesity, there are still many gaps in this field. This Research Topic attempts to collect work on the role of circadian rhythms in obesity in effort to provide additional insight toward the development of novel strategies that prevent and/or treat obesity related circadian dysfunction.

Three papers of this Research Topic focused on the role of sleep among those with obesity. Tang et al. provides a narrative review on the impact of obstructive sleep apnea on nonalcoholic fatty liver disease (NAFLD). Given that nearly 25% of the global population have excess fat in the liver and many struggle to get adequate sleep, attention towards factors raising cardiometabolic risk documented in people living with fatty liver is needed. In turn, the review provides mechanistic insight into how disrupted sleep impacts systemic physiology relates to cardiometabolic health. This is of clinical significance in providing targets for preventing and exploring potential therapeutic targets for NAFLD as it relates to sleep.

Expanding the scope to sleep, Ren et al. studied the association of sleep duration with body mass index (BMI) in over 3000 individuals from rural China. Importantly, more attention is being placed on potential sex differences promoting cardiovascular disease rates between mid-life and later adulthood (6). The factors promoting such divergence among men and women is unclear. However, U-shape relations (too much or little) of sleep duration has been a factor considered to potentially drive chronic disease risk. Herein, the authors interestingly report that relationships between sleep duration and BMI are sex-dependent, whereby women sleeping toward 9h have lower BMI while men near 8h have high BMI. Together, this suggests an impact of sleep duration on obesity may be sex specific, and additional investigation is required to confirm preventive approaches to obesity.

Napping is of potential interest to balance a lack of sleep for health purposes. In this Research Topic, Zambrano et al. addressed the effect of habitual napping on circadian rhythms of lipase E (*LIPE*), an encoding enzyme of hormone-sensitive lipase (HSL), in abdominal adipose tissue. HSL plays an important role in initiation of lipolysis. Interestingly, and perhaps somewhat surprisingly, the authors demonstrated in people well matched in key clinical demographics as well as total sleep and meal frequency that *LIPE* expression and HSL activity displayed blunted circadian rhythms in nappers compared with non-nappers. This suggests that napping itself or the need to nap per se may alter lipid mobilization and contribute to altered fat tissue function in habitual nappers. The study confirms the important role of the sleep-wake pattern in metabolic relations, providing novel molecular insights.

Shifting the focus to light at night as a potent disruptor of the circadian system, Zhang et al. investigated a large nationally representative sample of 98,658 adults from 162 study sites across mainland China. The study revealed significant associations of light at night exposure with prevalent obesity in each sex and age category, especially in men and older people. Together, the findings suggest that reducing light exposure at night might be a public health effort considered in obesity prevention.

Lastly, the role of meal timing as gained interest in recent years (7), as later day eating is associated with disease risk, particularly among people who identify has later chronotypes (e.g. night owls) (8). A comprehensive review of Peters et al. highlighted the association of mistimed food intake with disturbed circadian rhythms and increased risk for obesity. Further, it was suggested that meal timing that aligned with endogenous circadian rhythms can optimize metabolic functions. Additionally, the paper explored physiological, behavioral, cultural, and environmental factors influencing meal timing. Thus, the review sheds light on crucial dietary factors that should be considered in guidelines for effective lifestyle modifications to prevent and combat obesity.

Taking together, the articles from Tang et al., Zambrano et al., Zhang et al., Ren et al., and Peters et al. provide a valuable contribution to understanding the role circadian rhythms in obesity development as well as provide further evidence for the need for more research in the field of chronobiology to optimize treatments designed to prevent/treat chronic disease.

## Author contributions

OP-R: Writing – original draft, Writing – review & editing. SM: Writing – review & editing.
